# Exploring the Lived Experiences of Instrumental Ensemble Performers With Dalcroze Eurhythmics: An Interpretative Phenomenological Analysis

**DOI:** 10.3389/fpsyg.2020.00336

**Published:** 2020-03-03

**Authors:** Catrien Wentink, Liesl Van der Merwe

**Affiliations:** MASARA, School of Music, North-West University, Potchefstroom, South Africa

**Keywords:** lived experiences, instrumental ensemble performers, Martinů, Dalcroze Eurhythmics, interpretative phenomenological analysis, experiences of flow

## Abstract

The purpose of this interpretative phenomenological analysis (IPA) is to explore the way that instrumental ensemble performers understand their experiences of Dalcroze Eurhythmics while preparing the Tango and Charleston of the jazz ballet *La revue de Cuisine* by Bohuslav Martinů. The participants of this study were the members of an *ad hoc* ensemble, which included both authors. Data included semi-structured interviews and reflections by the first author during and after the five Dalcroze sessions. The six emergent super-ordinate themes were: (1). Heightened awareness of music, time, space and energy; (2). Beneficial for relationships in the ensemble; (3). Improved musicianship; (4). Enjoyment and feeling good; (5). Informing pedagogy; and (6). Social and cognitive challenges. Implications of this study suggest that Dalcroze experiences link with Csikszentmihalyi’s description of the experience of “flow.” Experiences of flow are very positive emotions, which will be beneficial for the performers in any instrumental ensemble.

## Introduction

The purpose of this interpretative phenomenological analysis (IPA) was to explore how instrumental ensemble performers understood their experiences of Dalcroze Eurhythmics while they were preparing the Tango and Charleston of *La revue de Cuisine* by Bohuslav Martinů. Juntunen (2004, p. 15) defined Dalcroze Eurhythmics as “an approach to music education that builds on the ideas of Emile Jaques-Dalcroze” and as such it can be seen as a “process for awakening, developing and refining innate musicality through rhythmic movement, ear-training and improvisation … that focuses awareness on the physical demands of artistic performance” (Carnegie Mellon University, 2019).

The three principal branches of this approach are rhythmics, solfége and improvisation (Le Collège de l’institut Jaques-Dalcroze, 2009, p. 4). According to *The Dalcroze Identity* (Le Collège de l’institut Jaques-Dalcroze, 2009, p. 8), “Rhythmics is at the core of Dalcroze education” and is not applied only in the rhythmics class, but to the teaching of the other branches as well. In solfége the awareness of pitch and relationships between pitches is heightened as well as the memory of different timbres (Le Collège de l’institut Jaques-Dalcroze, 2009, p. 13). Lastly, “improvisation is one of the most important abilities or tools” in Dalcroze Eurhythmics (Le Collège de l’institut Jaques-Dalcroze, 2009, p. 17). The teacher uses improvisation for different activities and the students improvise in response to the teacher’s improvisations. Nivbrant Wedin (2015, 217) described this approach as entailing “an attitude, a way of thinking.” Juntunen (2004, p. 17) defined Dalcroze Eurhythmics as an example of the way to ground music teaching in embodied experiences.

Integrating Dalcroze Eurhythmics into ensemble playing can have valuable results for instrumental ensemble musicians preparing for a performance, because performing is one way to embody music. Another important aspect of Dalcroze Eurhythmics is group work, including the mutual interactions between group members. Jaques-Dalcroze’s intent was to develop a sense of self as well as a sense of sociability through his exercises. In the exercises students worked alone, in pairs and in groups (Habron, 2014, p. 94; Nivbrant Wedin, 2015, p. 231). This focus on group work is another reason why Dalcroze Eurhythmics could be of great value to an ensemble preparing for a performance.

The problem driving this research is performer-orientated and has three dimensions: a real-life problem for us as ensemble players is listening to our co-performers while playing in an ensemble; a second dimension is the limited time instrumental ensemble performers often have to learn new music and perform it as an ensemble; and the third aspect is the limited research available on the experiences of ensemble players who use Dalcroze Eurhythmics to prepare for performance. The first aspect is a real-life problem which we experience as performers in the ensemble, namely that we often find ourselves in a situation where we are so focused on our own playing that we do not really listen to what our fellow musicians are playing. Especially in chamber music, it is absolutely essential for performers to listen to each other. Edwards (2014, p. 26) noted that many chamber groups “play with blinders on.” He stated that they “zoom in” on their own notes and are completely oblivious to what is going on in the rest of the ensemble.

The second part of the problem is that instrumental ensemble performers often have a large number of compositions to master in the ensemble and little time in which to do this. Dalcroze Eurhythmics is an approach that can possibly be used to help overcome this problem, as it helps one understand the music and incorporate the rhythms and phrases into one’s body. In research conducted by Van der Merwe (2015, p. 399) on the experience of music students with Dalcroze-inspired activities, most of the participants acknowledged that the bodily experience facilitated their understanding of the music. A study by Habron et al. (2012, p. 35) on the experience of student composers with Dalcroze Eurhythmics produced similar results, with two thirds of the participants that stated the Dalcroze Eurhythmics expedited their learning process. Juntunen and Westerlund (2001, p. 210) argued that Dalcroze exercises could have an impact on various other abilities such as focusing attention and heightening awareness, memory and concentration as well as enhancing the ability to pick up a habit quickly.

The third aspect of the problem is the limited amount of research on the experiences of instrumental ensemble performers who use Dalcroze Eurhythmics. Research has been done on the application of Dalcroze Eurhythmics in ensembles. In choral music preparation Dalcroze Eurhythmics was used by conductors, as a rehearsal strategy, to help choirs prepare for performances through movement (Chagnon, 2001; Daley, 2013). Crosby (2008) wrote an article on using Dalcroze activities in choir rehearsals. He developed his own teaching strategies to incorporate movement into the learning process for choirs as this method helps with students’ rhythmic internalization, breath energy and phrasing. Henke (1984, 1993) used Dalcroze as a rehearsal technique in choral and band rehearsals. Our study is different from these research projects as its focus is on the experiences of instrumental performers in an ensemble with Dalcroze Eurhythmics while they were preparing for a performance.

Although no studies have been conducted on the experiences of instrumental ensemble performers, there are studies on teachers and students’ experiences of Dalcroze Eurhythmics. Studies were conducted on the experiences of music education students (Van der Merwe, 2015), composition students (Habron et al., 2012) and conducting students (Marzuola, 2019) using Dalcroze Eurhythmics. Alperson (1995) discussed the experiences of adults with Dalcroze Eurhythmics in her thesis. Juntunen’s (2002) study was on the experiences of master Dalcroze teachers in facilitating this approach, while Dutton (2015) explored the holistic experiences of Dalcroze teachers and students in Dalcroze pedagogy.

The only three studies that discussed the experiences of instrumental performers with Dalcroze Eurhythmics are those by Greenhead (2016, 2017) and Ridout (2019). Greenhead (2016) wrote about a violinist’s experiences with Dynamic Rehearsal^1^ and the experiences of performers when Dalcroze principles were applied to the rehearsal and performance of musical repertoire (Greenhead, 2017). She found that the performer experienced transformation, being able to approach the music in a new way (Greenhead, 2016). Furthermore, the performers became the music, their sound became more resonant (Greenhead, 2017). Ridout (2019) studied three flute players’ lived experiences of Dalcroze Eurhythmics in preparing contemporary music for performance. She found that the body provides a way into contemporary music, facilitates a deeper knowledge and connection to the music and enhances communication with the audience. Ridout’s (2019) study is similar to ours, since it is also an interpretative phenomenological study. However, our study differs from hers because we studied the experiences of an ensemble that prepared for performance and not only individual experiences. No further studies that we are aware of were conducted on the experiences of instrumental ensemble performers with Dalcroze Eurhythmics. Therefore, the research question that guided this study was: How do ensemble performers understand their experiences of using Dalcroze Eurhythmics to prepare for a performance of the “Tango” and “Charleston” of the jazz ballet La revue de Cuisine by Bohuslav Martinů?

## Procedures

The research design used in this study was a qualitative design and investigated how the participants in an ensemble understood and interpreted their own experiences (Creswell, 2013:43) of using Dalcroze Eurhythmics for performance preparation. Therefore, this study was not a quantitative intervention that measured and compared the value of Dalcroze Eurhythmics with a “before” and “after” version of the performance. In this qualitative study we followed an interpretative phenomenological analysis (IPA) approach (Smith et al., 2009, p. 1), which explored how instrumental ensemble performers made sense of their lived experiences with Dalcroze Eurhythmics. IPA originated from three key areas of knowledge construction: hermeneutics, phenomenology and idiography (Smith et al., 2009, p. 1). Our research followed the creative approach of boundary pushing (Coyle and Olsen, 2011, p. 174). We worked within the boundaries of IPA, but the boundaries were pushed in the sense that we included our own experiences in this study. This was a new approach to IPA, but as Larkin (2015), co-writer of the handbook on IPA, stated in a personal e-mail to us on 10 February 2015: “It is unusual, but it can be done and it isn’t in conflict with the core conceptual underpinnings of IPA.”

### Role of the Researchers

As already mentioned, our roles encompass being members of the ensemble who actively participated in the Dalcroze sessions and included our own experiences as data. The researcher in IPA research has to understand and interpret the participant’s experiences from an insider’s perspective (Smith and Osborn, 2008, p. 53); in our case we were insiders, which gave us some extra insight into the experiences of the other participants. However, the researchers were not the Dalcroze teachers, but were participants in the ensemble and research. Being participants in the research gave us the opportunity to reflect on our experiences.

Finlay (2002, p. 209) wrote several articles on reflexivity and, according to her, there are five variants of reflexivity: (1) “introspection”; (2) “intersubjective reflection”; (3) “mutual collaboration”; (4) “social critique”; and (5) “discursive deconstruction.” Our research falls primarily into the third category, mutual collaboration. In this type of research, the researchers are simultaneously participating in their own research and they engage in circles of shared reflection and experience with the other participants (Finlay, 2002, p. 218).

As the researchers we had various roles. The first author conducted semi-structured interviews with six of the participants (also with the second author) after noting her own reflections. It is important to point out that she first wrote down her own reflections on the semi-structured interview questions before any interviews with participants were conducted. She did this to make sure that her own reflections were her personal experiences and not influenced by what she heard in the interviews. This process of writing down own reflections, resonates with the practice in phenomenology called bracketing (Merriam and Tisdell, 2016, p. 27), where the researcher temporarily set aside personal views in order to be open to the participants’ experiences and, in this case, to be honest about her own experiences. Both authors analyzed and interpreted the data from the six interviews as well as the reflections from the first author. Therefore, in the introduction and discussion sections of this article we speak as researchers, but in the presentation of the findings our experiences are included as data.

### Participants

According to Smith et al. (2009, p. 51), there are no right answers to the question of sample size and for doctoral research one can use up to eight participants (Smith et al., 2009, p. 52). Reid et al. (2005, p. 22) stated that ten participants are at the higher end of sample sizes for IPA. Therefore, our sample size of seven participants, is at the higher end of what might be deemed appropriate for an IPA study.

*La revue de Cuisine* is a sextet consisting of violin, cello, trumpet, clarinet, bassoon and piano. Judy^2^ was the violinist, Peter the cellist, Anne the trumpet player, Mary and Benjamin were the two clarinet players who attended different sessions, Emma was the bassoonist and Cathy the pianist. The clarinet player had to be substituted with a different player in the last three Dalcroze sessions, but we decided to use the interviews of both clarinet players as data. Therefore we used the interviews of six ensemble members and the reflections of the first author, making it seven interviews The participants were two males and five females. This is a homogenous sample since all the participants are music teachers and instrumental performers. Table 1 mentions the Dalcroze and dance experience of the seven participants. To ensure that all the findings were trustworthy, we used the strategy of member checking for validation (Creswell, 2013, p. 252; Merriam and Tisdell, 2016, p. 246). The researchers sent the analysis of each participant to them and asked them if they were understood correctly. Feedback from the participants were then incorporated into the findings. Although the findings are not generalizable in a statistical sense, the reader can decide to what extent these findings are transferable to their own contexts. Furthermore, this study contributed to theory development with the link between Dalcroze experiences and the flow experience (see section “Theoretical and Practical Implications of This Study: Flow”).

**TABLE 1:** Prior Dalcroze and dance experience of participants.

**Most experience**	**Some experience**	**Littleexperience**	**No experience**
**Emma (Bassoon)** • Dalcroze experience • Dance experience (12 years) • Did perform the Martinů work already	**Cathy (Piano)** • Dalcroze experience • No dance experience	**Peter (Cello)** • Dalcroze experience • No dance experience	**Mary (Clarinet)** • Clarinet in session 1 and 2
**Judy (Violin)** • Dalcroze experience • Dance experience (12 years)			**Benjamin (Clarinet)** • Clarinet in session 3,4 and 5 • Did perform the Martinů work already
			**Anne (trumpet)** • Did perform the Maritnu work already.

### Data Collection and Analysis

The main approach to data collection in IPA studies is the semi-structured interview (Eatough and Smith, 2008, p. 188), which was used in this study. There were two rounds of semi-structured interviews. The first interviews were conducted after the first two Dalcroze sessions and the second round of interviews was conducted after the last three Dalcroze sessions. The first author included her own reflections, made after the Dalcroze sessions as her own “interview,” before interviewing the other participants.

The process followed in IPA data analyses entails organizing, coding, integrating and interpreting of data (Reid et al., 2005, p. 22). The researchers used ATLAS.ti 7, a computer assisted qualitative data analysis program, to organize and analyze the data. We used the transcribed interviews as primary documents. Code filters were useful to keep the cases of the different participants separate. We followed the six steps listed by Smith et al. (2009, p. 82–107) in conducting this IPA data analysis. The first four steps take place with the data of each individual’s case separately, namely: (1) immersion in the data; (2) initial note taking; (3) developing emergent themes; (4) looking for links within the emergent themes. After the data analysis has been completed in a single case, step five entails repeating steps 1 to 4 with the next case (Smith et al., 2009, p. 100). The final step is looking for patterns across cases in the cross-case analysis (Smith et al., 2009, p. 101). In this study we first analyzed each participant’s interview separately by reading through the interview thoroughly, analyzing the data for codes and categories and developing emergent themes for the specific participant. After each participant was analyzed individually we looked for connections in the themes across cases and developed our super-ordinate themes.

### Ethics

Each participant was asked to complete an informed consent form that gave us permission to use the information obtained in the interviews in our research. The participants in our study were adult instrumental performers. This study did not render this population vulnerable as the participants were adult musicians who practise and play in ensembles as part of their daily lives. The participants were not harmed in this research and had the right to withdraw from this study at any stage. Their privacy was protected in the research through the use of pseudonyms. To protect the privacy of all the participants, it is not disclosed which of the participants were the authors.

### The Dalcroze Sessions

We do not have Dalcroze teachers in South Africa, but we had an opportunity to conduct five workshops when two qualified Dalcroze teachers^3^ visited the North-West University on three occasions. During the Dalcroze sessions only two movements, the Charleston and the Tango, from *La revue de Cuisine* by Bohuslav Martinů were used. *La revue de Cuisine* was written in 1927 as a jazz ballet for a sextet (piano, violin, cello, clarinet, bassoon, and trumpet) in ten movements, but the suite version of this ballet music (premiered in 1930) has just four movements. Since we had musicians available to meet the requirements of the orchestration of Martinů’s jazz ballet, we chose this work for our study. We only used two movements, the Tango and Charleston, as our time was limited.

It was not only the availability of the musicians and time limits that influenced our repertoire choices, but also the particular difficulties in the movements. Difficulties in the Tango include the switching between irregular note groups (triplets in quavers and crotchets as well as sextuplets) and regular note groups. These types of rhythms also need to be synchronized between instruments, which makes it difficult for an ensemble. In the Charleston, there are syncopated rhythms and difficult accentuated patterns. Hemiola is also found as the phrasing over bar lines and patterns interferes with the feeling of a regular time signature and also a clear first pulse. All these aspects can easily be addressed in Dalcroze Eurhythmics and therefore this work and these movements were suitable. The activities of these five sessions and the length of each session are included in Supplementary Table A, but we will highlight some of the activities that we did during these five sessions in the following section.

#### Session 1 – Dalcroze Teacher 1

We started this session with general Dalcroze Eurhythmics activities like walking the pulse and showing the pulse in different body parts. Some other general activities that we did were focused on the ensemble. We walked in the room (without music) and had to try and find a unified pulse in our walking, between the whole ensemble. We also listened to recorded music while clapping the pulse and passing the pulse around to other ensemble members. When passing the pulse around you had to be very clear and indicate to whom you were passing the pulse through eye contact. After this we worked on the Charleston. First we improvised and did any free movement on the complete Charleston. After this we listened to the opening of the Charleston with closed eyes and showed the melody line with our hands. For the next activity the Dalcroze teacher asked us to show the cello line and then the bassoon line with our hands. Then we moved freely on the opening of the Charleston. Subsequently, we had to portray the cello line followed by the bassoon line with improvisation and free movement. We paired up and one person would portray the bassoon line and another person the cello line. We also changed partners and lines during this activity.

#### Session 2 – Dalcroze Teacher 2

In this session we started with general activities that were focused on tuning in on each other as an ensemble. In the first activity the teacher played one voice, two voices or full chords and we had to walk alone (one voice), in pairs (two voices) or together as an ensemble (full chords). After this we did an activity where everyone had to invent their own interesting walk and pair it with a sound. On signals from the teacher we had to start merging our walk and sounds. First in pairs, then pairs merged and finally we had to morph our walk and sound into one unanimous walk and sound as an ensemble. We then moved on to an activity where fast recorded music was played and we had to travel through the room with a scarf. You could use different methods to carry the scarf. The same fast recorded music was used, but now we used one ball and traveled through the room with the ball, passing the ball along to any other ensemble member at a suitable place in the music, like a phrase ending.

In the next activity we listened to the complete Tango and the teacher handed out a piece of paper and markers for each ensemble member. The teacher divided the Tango into five smaller sections. We had to draw a stage setup of where we see all the instruments in each of the five sections. You had to indicate where on a big stage you would place the different instruments during that section of the Tango (what is foreground, background etc.). Then we discussed our drawings with each other and started creating a stage setup and a choreography of the Tango. Each person portrayed their own instrument. In the end we performed the choreography of the complete Tango.

#### Session 3 – Dalcroze Teacher 2

We started this session by discussing the background of the Tango. Then we did some general warm up exercises. Afterwards we portrayed the characters of different types of music through improvisation before we did some improvisation and free movement to the sound track of the Tango. Then we picked up our instruments and played through the complete Tango. After this the teacher did a sequence of activities that lead to the synchronization of two against three pulses through movement. We stood in a circle and walked three pulses, while clapping two pulses and then switched, putting the three pulses in the hands and the two pulses in the feet. The teacher played three-note patterns or two-note patterns on the piano. First you had to walk to the pattern being played and after that you had to walk to the opposite pattern of the one being played. We ended this session by moving back to the instruments and now playing three note-patterns and two-note patterns on the pentatonic scale on our instruments. You could choose whether you wanted to play the two-note pattern or the three-note pattern and you could switch between them at any time. We had to close our eyes and try to listen to other instruments playing the same pattern. We had to make sure that we were together and synchronized our patterns. Then we chose two and three note patterns from the Tango and performed it together a few times.

#### Session 4 – Dalcroze Teacher 2

The Tango starts with two opening chords. Everybody was given a ball and each person had to portray their own version of these opening chords through bouncing, catching, throwing or any other movement with the ball. We showed the different versions to each other and then performed each version on our instruments, while the teacher was showing the different versions with the ball. In the end we had to decide which version we preferred as an ensemble and then we practised that version, on our instruments. Then we played through the Tango again and started working in more detail on specific rhythms and passages that we were struggling with. The cellist had a big solo in the beginning of the Tango with complicated rhythms and an ostinato accompaniment by the piano. The pianist started tapping this rhythm on the back of the cellist to help him fit his solo rhythms into this ostinato pattern correctly. We ended this session by discussing how we see the characteristics of the different instruments in this Tango.

#### Session 5 – Dalcroze Teacher 2

We started our last session by playing through the Charleston first. The next activity we did was to stand opposite each other in groups of three. The teacher played a recorded track of “Take five” and we walked toward each other, but were only allowed to walk on the pulses that the teacher called out. In the next activity we paired up with a partner and had to choose eight different quick movements and put them in a specific order. After we practiced our movement sequence, the teacher played eight pulses on the piano and called out any number from one to eight. We had to perform all the movements *pianissimo* and quietly, but the movement from the number that was called out should be performed *fortissimo* and big. Then we started putting the accents on the movements in a very specific order that was derived from a specific passage in the Charleston. We practiced this pattern with our eight movements, then started walking toward each other, only stepping on the accentuated notes in this practiced pattern. We picked up our instruments and started performing this accentuated pattern, first on one note. Next we played the specific pattern from the Charleston, first at half speed and then we moved the tempo up. We had to focus on the places where the accents should be placed in this specific passage.

## Findings

**FIGURE 1:**
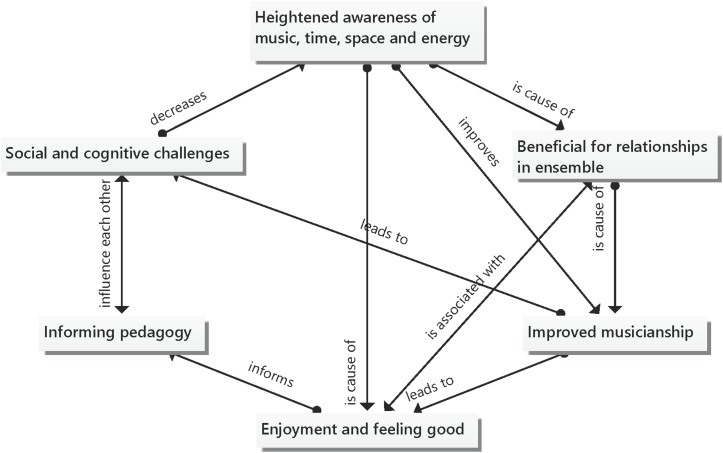
Super-ordinate themes that emerged from the cross-case analysis.

During the cross-case analysis (see Supplementary Table B) six interrelated super-ordinate themes emerged from the interviews and reflections of the seven participants (Figure 1). The Dalcroze sessions led to heightened awareness of music, time, space and energy for the participants. This heightened awareness promoted awareness between the ensemble members, which was beneficial for relationships in the ensemble. This in turn led to an improvement in the musicianship of the ensemble members. When musicianship improved, the participants felt more accomplished and less self-conscious, which in turn led to enjoyment and feeling good. Learning is enhanced when ensemble members experience enjoyment during rehearsals. This important role that enjoyment plays in learning informs pedagogy. Although the response and feedback were largely positive, participants did experience social and cognitive challenges as a result of a Dalcroze teachers’ teaching styles, this too can inform Dalcroze teachers’ practices and pedagogy.

### Super-Ordinate Theme 1: Heightened Awareness of Music, Time, Space and Energy

The first super-ordinate theme that emerged was the heightened awareness of music, time, space and energy (see Supplementary Table B). The heightened awareness of music included three different emerging aspects: awareness of general musical aspects, awareness of other players on a more personal level, and awareness of the different interactions within the ensemble. Regarding the awareness of musical aspects, Cathy mentioned that she became more aware of the accents in the Charleston, Mary noticed the phrasing and characters in the music, and Peter became increasingly aware of the dynamics, rhythm and tempo in the music. Cathy was aware of the moods of the other ensemble members. The heightened awareness of music also had to do with the different interactions within the ensemble. All of the participants, except Benjamin, talked about hearing the other parts and the interaction and role of the other instruments differently after the sessions. On this issue Mary said: “You know you listen to a piece in one way, you know only in relation to your own instrument and with both these sessions, you were made aware of the other instruments and understanding their melodies better.” Benjamin thought there was a heightened reciprocal interpersonal awareness and, according to him, that helped with enhancing the sense of togetherness in the ensemble.

The other three aspects in this first super-ordinate theme were the heightened awareness of time, space and energy. All the participants except Benjamin discussed their awareness of the passing of time during these sessions. Overall the participants experienced the passing of time as being quicker in the sessions they were actively involved in (Judy, Anne, Cathy, Emma), or did a lot of different activities in (Anne, Judy) and worked in the group more (Anne, Mary). The moment that the participants felt that the focus was too much on the individual, they experienced the time as passing slowly (Emma, Anne). Another aspect that made the passing of time seem slower was when the teacher lingered too long on one activity (Emma, Anne, Cathy) and when the activities were too challenging (Emma, Mary). Emma explained why she experienced the passing of time differently in the different sessions: “Where I struggled, it felt like everything was going in slow motion and then it wasn’t a nice place for me to be in. So where it was easy, the time flies, and where it was difficult, it felt like forever.” The time thus passed quicker in activities where the participants were actively involved and if the activity was not too challenging. When the focus shifted to individuals and not group work and where the activities were too challenging or lingered too long, the passing of time slowed down for the participants.

Regarding the awareness of space, all the participants except Benjamin talked about how they experienced the utilization of space during these sessions. One aspect that emerged in all the interviews was that the space used for movement felt bigger when working together as a group, while with individual work and work in pairs, it felt like less space was covered. Kathy and Emma talked about the awareness of energy in their interviews. They mentioned that they enjoyed the energy in Dalcroze sessions. Emma noticed that in one of the activities we did with balls, the energy we portrayed with the balls was transferred to the music when we started playing again. Furthermore, the participants became more aware of each other and of the different relationships in the ensemble.

### Super-Ordinate Theme 2: Beneficial for Relationships in Ensemble

All the participants experienced the Dalcroze sessions as being beneficial for the relationships in the ensemble (see Supplementary Table B), especially in three respects: bonding and building relationships, promoting group dynamics, and the freedom to express different interpretations during these sessions. All the participants stated that the sessions acted as a good icebreaker and really helped the ensemble to bond quickly. Peter said that it helped him to feel comfortable more quickly in the new ensemble. Benjamin stated that it was really good for teamwork: “this kind of activity, I think, could build the teamwork that would make the musical attention to each other happen more quickly.” According to Emma, Cathy, Anne, Judy and Benjamin, this bonding goes a bit deeper and can build relationships of trust in an ensemble. To bond, build trust and feel more comfortable in an ensemble more quickly, will be very valuable for any ensemble.

All the participants except Benjamin discussed some aspects that could promote group dynamics. Judy thought that the ensemble members were respectful toward each other in their movement improvisations. Mary also felt very strongly about the fact that the Dalcroze sessions promoted verbal and musical communication in the ensemble. Emma and Mary posited that these sessions could further help an ensemble with conflict resolution. Mary said: “Dalcroze is an incredible way of conflict resolution in an ensemble, but then all the parties need to commit.” It is very valuable that Dalcroze could contribute to group dynamics, mutual respect, promote communication and facilitate conflict resolution in this ensemble.

The third aspect of this super-ordinate theme that emerged from the interviews was the different interpretations from different combinations of people in the ensemble. As Judy explained: “you bring your own voice and kind of like your own interpretations in what’s going on and I think we can learn a lot from how we, how our fellow ensemble players move and think.” Peter and Judy stated that the Dalcroze sessions gave them different views on the possible interpretations when performing the Martinů.

### Super-Ordinate Theme 3: Improved Musicianship

In the third super-ordinate theme, improved musicianship (see Supplementary Table B), four different aspects of improvement emerged: musical understanding of the Martinů, sound production, musicianship, and ensemble playing. Cathy, Peter and Judy stated that the sessions improved their musical understanding; as Cathy clarified: “I didn’t know the Martinů work very well beforehand and it really helped to get to know the piece better.” Anne thought her musical understanding had improved as the sessions gave her a more holistic picture of the Tango.

Some participants thought that these sessions and previous exposure to Dalcroze Eurhythmics improved their sound production (Emma, Judy), while Cathy said that Dalcroze training had an influence on her piano touch. According to Peter: “the moment that you are aware of your body and you are relaxed, you play ten times better and I think Dalcroze and movement to music accelerates that process.” The Dalcroze Eurhythmics sessions helped him to be more relaxed when playing the cello and in effect improved his sound and playing.

More aspects that led to the improvement of musicianship included having a better idea of how to practise correctly (Peter) and improved listening (Anne, Judy, Mary). Mary said that: “It definitely sharpens your listening and your awareness of other musicians.” Being more self-confident also improved the participants’ musicianship (Cathy, Anne, Peter). Cathy, Anne and Peter talked about how Dalcroze Eurhythmics helped them with their self-confidence when performing and to overcome their self-consciousness during the Dalcroze sessions. Cathy, Judy and Emma indicated that Dalcroze Eurhythmics improved and developed their musicianship: “I think Dalcroze helped to develop me as a musician […] it deepened my experience of the music, it expanded my expressive possibilities and it reduced my inhibitions” (Emma).

Cathy and Benjamin mentioned that the sessions improved the synchrony of the ensemble and in this regard Emma stated that the clarity of the ensemble playing improved. She experienced these sessions as improving the ensemble’s interpretation of the work. Anne had similar feelings about this: “and you could definitely see, if we went back to the instruments, that there was a big difference. So that was very positive for me, it was almost like a before and after.” Overall the sessions were experienced as being beneficial for the ensemble and individuals as it improved their self-confidence and musicianship and thus led to better ensemble playing.

### Super-Ordinate Theme 4: Enjoyment and Feeling Good

From the interviews and reflections, it became clear that all the participants really enjoyed the Dalcroze sessions (see Supplementary Table B), which enhanced the general sense of happiness for certain participants. All the participants enjoyed the group work most and for Cathy it really inspired her “to move with others.” Anne and Judy enjoyed activities with equipment such as balls or scarves, while Cathy’s enjoyment increased with activities that felt familiar to her. Peter enjoyed the rhythm activities, which felt like a game, and when there was a clear goal: “where we had to improvise movements on that series of numbers that we walked on. The things where we had a goal, I enjoyed the most.” Peter and Judy experienced the adding of instruments in the last three sessions as positive and for Peter “the ultimate was the mixture between playing and moving.”

For many participants the enjoyment went a bit deeper and led to a greater sense of feeling good. Emma and Cathy experienced Dalcroze Eurhythmics as inspiring and it gave them energy. Emma and Judy liked the freedom that they experienced in Dalcroze classes. Judy and Peter refer to the fact that these sessions improved their mood and Mary felt that the focus on music can be really therapeutic: “You are forced to leave whatever negative or positive, or what issues you have concerning your good or bad day, and you are focused to listen to the music and to react to that, and that is I think very good therapy for getting your mind clean.” Enjoyment and feeling good were thus important aspects that emerged in all the interviews and this can improve the mood of the whole ensemble.

### Super-Ordinate Theme 5: Informing Pedagogy of Music Educators and Dalcroze Practitioners

The fifth super-ordinate theme, “informing pedagogy,” emerged in all the interviews (see Supplementary Table B). All the participants are also music teachers. This explains why they made suggestions about pedagogy through their observations and experiences. Four aspects emerged in this fifth super-ordinate theme: personal preference is important, you must experience Dalcroze yourself, the teacher has an important role, and learning through movement is a feasible approach.

Personal preference is important in this approach to preparation and even though most of the participants felt very positive about it, Benjamin argued that he did not really learn anything and has doubts about the value of the approach. This confirms the observations of Cathy and Emma that this type of preparation might not work for everybody: “I do think that not everybody enjoys Dalcroze. but I have not met many people who didn’t enjoy it after doing it” (Cathy). Emma and Mary felt very strongly that you must experience Dalcroze to truly understand it and that it cannot really be explained to somebody through words.

Emma prefers a learner-centerd approach. “If I become self-conscious, it becomes a nightmare. So I appreciate it if a facilitator moves the attention away from the individual” Benjamin struggled to move with the right foot first because “in dancing for a man that’s the opposite way round, and if you’re in the army and you’re marching it is left foot first also, so I was always being caught wrong-footed.” To be flexible to learner preferences and not put students on the spot are important principles for Dalcroze practitioners to consider.

If the facilitator handles the sessions in a constructive and positive way, then learning through movement can be a very feasible approach (Emma, Cathy, Anne, Mary). According to Emma and Cathy, learning through movement is a fun way to learn and also the best way to learn. Anne and Peter, on the other hand, thought that even though it is a feasible approach, it is most appropriate for first-time players of a piece or new ensembles. Peter said “This is my view, but I think the more amateur the people in the ensemble are, the more definite the difference will be.” Another aspect that can inform pedagogy and teaching strategies for teachers is the social and cognitive challenges that participants experienced in these sessions.

### Super-Ordinate Theme 6: Social and Cognitive Challenges

The last super-ordinate theme that emerged in all the interviews was the challenges (see Supplementary Table B) that the participants experienced during these Dalcroze sessions. These challenges were either social, cognitive or physical. Regarding the social challenges, Cathy, Anne, Peter and Mary mentioned that they felt self-conscious during some activities in these sessions. Cathy, Anne and Peter indicated that free movement made them feel self-conscious: “I do not like the part of Dalcroze where you have to move around randomly and freely […] I am not a contemporary dancer and it doesn’t really help me to understand the music.” (Peter). Mary’s self-consciousness emerged from her own “awkward body issues.” Three of the participants, talking about self-consciousness, were very new to Dalcroze.

The cognitive challenges experienced by the participants can be divided into two issues: separating music and movement, and confusion. Emma, Cathy and Benjamin experienced this separation between music and movement in some of the activities during the Dalcroze sessions; as Benjamin explains: “I found the movements themselves became something to have to learn. In that respect it was a negative experience because in my mind it took my mind away from the music.” The second cognitive challenge had to do with the confusion experienced by the participants. Peter and Emma felt confused when the activities were too abstract and did not seem to have a clear goal. Emma commented on an activity where she had to draw the music: “I was confused when we had to sit and draw. I couldn’t understand how I could put something that happens in time on a piece of paper.” Benjamin, the oldest member in the ensemble, was the only participant who experienced some physical challenges. He was physically sore afterwards and he struggled with his breathing when playing the clarinet after a movement activity.

## Discussion

The six super-ordinate themes that emerged through our data analysis are discussed and linked to relevant scholarly literature and implications for practice regarding these findings are given.

### Theme 1: Heightened Awareness of Music, Time, Space and Energy

All the participants mentioned one or more areas in which they experienced heightened awareness during the Dalcroze sessions. In our research, six of the seven participants mentioned that the Dalcroze sessions made them aware of their own parts in the ensemble, of their own part in relation to the other parts, and of the interaction between the various parts. According to Keller (2001, p. 35), the physical demands needed for an artistic performance are different for ensemble performers than for solo performers. Wenger (2006, p. 60) claims that a very complex skill that instrumental ensemble performers need to develop is to hear all the additional parts while still successfully performing their own respective parts. Dalcroze Eurhythmics can help an ensemble to become more aware of all the different parts and thus address the problem Keller (2001, p. 20) refers to as attentional resource allocation in ensemble performances. Juntunen and Westerlund (2001, p. 208) corroborate this: “The Dalcroze exercises help in solving the problem of unexpected information without losing the flow of the movement. Hence they prepare the musician to interact smoothly without interruption in changing musical situations.” Not only did Dalcroze Eurhythmics help instrumental ensemble performers to become more aware of the interactions between the different parts in the music, but socially they also become more aware of each other, which becomes clear in the discussion of the next theme.

### Theme 2: Beneficial for Relationships in Ensemble

The participants becoming more aware of each other in the Dalcroze sessions was beneficial for the relationships in the ensemble. Jaques-Dalcroze (1921/1967, p. x) himself said that “teachers should aim at furnishing them [students] with the means both of living their own lives and of harmonizing these with the lives of others.”

The participants felt that the Dalcroze sessions helped the ensemble members to bond quickly and get to know each other faster. Making these connections with people on a spiritual level will have a further positive influence on the personal relationships between ensemble members. According to Keller (2001, p. 25), the musicians in his study claimed that the enhanced personal relationships in the ensemble helped them to integrate better with the other musical parts in an ensemble. For Keller (2001, p. 25) this highlighted the value of approaches that examine interactions within ensembles. Moran (2011, p. 5) added to this observation by arguing that effective musical communication in an ensemble is dependent on the quality of the personal relationships between ensemble members.

### Theme 3: Improved Musicianship

One of the aims of Dalcroze teaching is to deepen musical understanding (Juntunen, 2016, p. 157). All of the participants in our study mentioned that the Dalcroze sessions improved their musical understanding of the piece. Some of the participants went a little further and stated that the Dalcroze sessions and Dalcroze Eurhythmics in general improved their musicianship. Juntunen (2004, p. 15) concurs that “Dalcroze Eurhythmics can be seen as a process for awakening musicality and developing musicianship in a broad sense.” For Greenhead (2016, Part 3, p. 5) personal knowledge and transformation (becoming a better musician and feeling more accomplished) “would lead to joy, feelings of freedom, agency and self-confidence.”

### Theme 4: Enjoyment and Feeling Good

Jaques-Dalcroze (1909/1920, p. 32) wrote: “I like joy, for it is life. I preach joy, for it alone gives the power of creating useful and lasting work.” The participants enjoyed the Dalcroze sessions. According to Juntunen (2004, p. 75; Juntunen, 2016, p. 254), Jaques-Dalcroze and master Dalcroze teachers wholeheartedly believed that joy is a very powerful stimulus for learning. This aspect of enjoyment that the participants experienced in our study can thus help ensembles to learn better and faster when preparing for a performance.

### Theme 5: Informing Pedagogy

Jaques-Dalcroze felt that aspects of wellbeing such as the self-assurance of students and feeling positive about themselves were important conditions for learning (Alperson, 1995, p. 191). In the interviews with our participants all of them talked about different aspects that can inform learning and thus pedagogy. Participants mostly felt that Dalcroze Eurhythmics is a very feasible pedagogical approach to use in preparing for performance. In Dutton’s (2015, p. 199) study, Hania stated that Dalcroze training should be included in mainstream education because “it provides a valuable artistic and kinesthetic learning foundation that students would not otherwise experience.” In the study by Habron et al. (2012, p. 30) the participants also recommend Dalcroze Eurhythmics as a valuable approach, but some of them mentioned that it might work better with undergraduate students and that it would work better if it is introduced from an early age, something that Benjamin mentioned in our study. Other similar recommendations were made by our participants. They felt that the approach was feasible, but had more of an impact on players who played the Martinů work for the first time. One participant mentioned that this preparation would work best for amateur ensembles.

### Theme 6: Social and Cognitive Challenges

“Dalcroze Eurhythmics works with the whole human being and therefore has the potential to be exposing” (Habron et al., 2012, p. 41). This is something that five of our participants experienced and discussed in their interviews. They especially talked about feeling self-conscious and exposed when they had to perform free and individual movements. Our participants’ self-consciousness was often linked with free movement. According to Nivbrant Wedin (2015, p. 227), a Dalcroze teacher should provide “freedom with frames,” which means that unclear or overly wide frames can generate frustration, and in effect, block progress (Nivbrant Wedin, 2015, p. 228). Nivbrant Wedin (2015, p. 228) mentioned that this is something to keep in mind especially with new Dalcroze students, which some of the participants were. These challenges, the participants experienced, can help Dalcroze teachers to understand their students’ experiences better and improve their teaching strategies.

## Theoretical and Practical Implications of This Study: Flow

According to Reid et al. (2005, p. 20), IPA is an inductive approach (it is “bottom up” rather than “top down”) where no hypothesis or prior assumptions are tested, but theories may emerge from the data. This was what happened in our research. The correlation of the findings with the flow theory emerged after the data analysis process has been completed. All the conditions of flow were experienced and mentioned in the interviews. The section below makes it clear that Dalcroze Eurhythmics has the potential to initiate the flow experience as we link our findings to Csikszentmihalyi’s (1997, p. 133) description of a flow experience. According to Csikszentmihalyi et al. (2005, p. 230): “Flow is a subjective state that people report when they are completely involved in something to the point of forgetting time, fatigue, and everything else but the activity itself. […] Attention is fully invested in the task at hand and the person functions at his or her fullest capacity.” An ensemble will benefit if the people in the ensemble function at their fullest capacity during preparation. In the following discussion the conditions of the flow experience as listed by Csikszentmihalyi (1997, p. 133) are linked to the direct words of the participants that emerged from the findings (also see Table 2).

**TABLE 2:** Linking quotes from our study with the conditions of the flow experience (Csikszentmihalyi, 1997, 133).

**Conditions of flow**	**Quote**
“Goals are clear – One knows at every moment what one wants to do.”	*“The things where we have a goal, I enjoyed the most.” (Peter) “So that gave us a unified goal, which helped a lot, because you struggle to agree on what the sound ideal of the ensemble should be, but with the ball exercise we reached unanimity easier.” (Emma)*
“Feedback is immediate – One knows at every moment how well one is doing.”	*“I could really hear that it helped the ensemble. Like in the “Charleston” it was much neater, actually amazingly clearer” (Emma)*
“Skills match challenge – The opportunities for action in the environment are in balance with the person’s ability to act.”	*“The challenges don’t seem impossible*…, *I never felt totally discouraged, which I think is great.” (Judy) “Let me explain it like this: if the challenge meets the ability, then time flies; if the challenge exceeds the ability, then time slows down.” (Emma)*
“Concentration is deep – Attention is focused on the task at hand.”	*“for that hour and a half that we worked together.you were so focused on what was going on. There was no time or energy or whatever to think about what was going on outside the room beforehand.” (Anne)*
“Problems are forgotten – Irrelevant stimuli are excluded from consciousness.”	*So you’re removed from your circumstances, that’s definitely because you have to focus so intently on your music [*…*] you can’t actually bring bad vibes or whatever into that session.’(Mary)*
“Control is possible – In principle success is in one’s hands.”	*“To walk on the rhythm and to clap, but mostly to walk on the rhythm. It is fun, because it really feels like you are getting it into your body.” (Peter)*
“Self-consciousness disappears – One has a sense of transcending the limits of one’s ego.”	*“After a few Dalcroze sessions moving together, then you know the people and then you aren’t shy anymore.” (Peter)*
“The sense of time is altered – Usually it seems to pass much faster.”	*“*…*it felt like five minutes. It was like oh is this now over, do we have to stop? In both sessions I can really say time flew by because I was having fun.” (Judy) “The time passed very quickly. I couldn’t believe that an hour had already passed when the session finished.” (Cathy)*
“The experience becomes autotelic – It is worth having for its own sake.”	*“If I was having a bad day.it takes you a little bit out of your situation and it removes you from your own reality, [*…*] You are forced to leave whatever negative or positive or what issues you have concerning your good or bad day, and you are focused on listening to the music and to react to that and that is I think very good therapy for getting your mind clean.” (Mary)*

The first condition of flow is that “Goals are clear – One knows at every moment what one wants to do” (Csikszentmihalyi, 1997, p. 133). In his interview Peter mentioned that he enjoyed the activities where we had a goal the most and Emma mentioned that the sessions “gave us a unified goal, which helped a lot, because you struggle to agree on what the sound ideal of the ensemble should be, but with the ball exercise we reached unanimity easier.”

The second condition of flow is that the “Feedback is immediate – One knows at every moment how well one is doing” (Csikszentmihalyi, 1997, p. 133). In the interviews Emma said that she could hear that it helped the ensemble immediately and that the Charleston was “much neater, actually amazingly clearer.”

The third condition of flow is that “Skills match the challenge – The opportunities for action in the environment are in balance with the person’s ability to act” (Csikszentmihalyi, 1997, p. 133). This condition of flow relates to the Dalcroze approach, because you can adapt the skill level for everybody and you can even adapt it while teaching if you see someone is struggling. In our study, Judy talked about the fact that the challenges did not seem impossible and that she never felt discouraged. Emma added to this condition by discussing her experience of time: “let me explain it like this: if the challenge meets the ability, then time flies; if the challenge exceeds the ability, then time slows down.”

The fourth condition is that “Concentration is deep and attention is focused on the task at hand” (Csikszentmihalyi, 1997, p. 133). In her interview Anne mentioned she was so focused on what was going on in that hour and a half session, that there was no time or energy to think about what was going on outside the room.

Being so focused links with the fifth condition of flow – “Problems are forgotten – irrelevant stimuli are excluded from consciousness” (Csikszentmihalyi, 1997, p. 133). Mary’s observation links with this condition directly: “So you’re removed from your circumstances, that’s definitely because you have to focus so intently on your music […] you can’t actually bring bad vibes or whatever into that session.”

The sixth condition of flow is about control that is possible and that “in principle success is in one’s hands” (Csikszentmihalyi, 1997, p. 133). For Peter it felt like he was taking control of his body when walking and clapping the rhythms and that it really felt as if he was getting the rhythm into his body.

The seventh condition of flow is “Self-consciousness disappears – One has a sense of transcending the limits of one’s ego” (Csikszentmihalyi, 1997, p. 133). Some of the participants mentioned that they did feel self-conscious during the sessions, especially with free improvisation, but Peter said after moving together in a few Dalcroze sessions he felt like he knew the people and he did not feel shy anymore. Similarly, Anne mentioned that she became less self-conscious as the sessions progressed and all the participants felt less self-conscious in group work.

The eight condition is about the sense of time that is altered and “usually it seems to pass much faster” (Csikszentmihalyi, 1997, p. 133). This was apparent in many of the interviews. Judy said “it felt like five minutes. In both sessions I can really say time flew by because I was having fun.” Cathy links with this by saying that she couldn’t believe that an hour had already passed when the session was finished.

The last condition of flow is that “The experience becomes autotelic – It is worth having for its own sake” (Csikszentmihalyi, 1997, p. 133). Even though there was a goal in these sessions regarding the ensemble works we had to learn, in the interviews it became clear that the experience itself, without the goal, was very valuable to many of the participants. Judy said: “Well I remember that Friday I was having a bad day. The moving made it so much better. I felt at peace.” Mary mentioned that it takes you out of your bad situation and removes you from reality.

As is evident in the above-mentioned quotes the Dalcroze sessions created an experience of flow for some of the participants during certain stages in the sessions. We do not, however, imply that all the participants experienced flow during all the Dalcroze sessions. The participants experienced the flow experiences as very positive. Csikszentmihalyi (1997, p. 137) explains that “people experience their own self as being stronger and more vital” after an experience of flow, which will boost the self-confidence of an ensemble. According to Nakamura and Csikszentmihalyi (2002, p. 244), this “flow state is intrinsically rewarding.” It causes a person to want to replicate the experience and will lead to personal growth in skills over time. The growth of skills will be rewarding, especially in a more amateur or newly constituted ensemble.

## Conclusion

The main research question guiding this study was: How do ensemble performers understand their experiences of using Dalcroze Eurhythmics to prepare for a performance of the “Tango” and “Charleston” of the jazz ballet La revue de Cuisine by Bohuslav Martinů? These experiences were synthesized in the six themes that emerged from the interviews. Through these six themes, possible solutions to the three dimensions of the problem that were discussed in the section “Introduction” were derived.

The problem was performer-orientated and had three parts, namely:

(1) A real-life problem for us regarding listening while playing in an ensemble;
(2) Limited time for performers to master new music and perform it in the ensemble; and
(3) Limited research available on the experiences of performers who use Dalcroze Eurhythmics to prepare for performance.

The first part of the problem was listening while playing in an ensemble. This has to do with Keller’s (2001:20) theory of attentional resource allocation. When focusing on your own part, you struggle to listen to the other instruments, and when you listen to the other instruments, you struggle to play your own part precisely. The Dalcroze sessions addressed this aspect for the participants and made them aware of the other parts and of the relationship between their own part and the other parts before starting to rehearse together. Some of the participants also felt that the sessions improved their listening and also their listening skills when the ensemble started playing together.

This aspect of improved awareness of the other parts and improved listening also addresses the second part of the problem regarding limited time for performers to master new music and perform it in the ensemble. Becoming more aware of the interaction between the different parts, before starting to rehearse together, can save time during rehearsals. Another aspect that could save time is the fact that the sessions helped the ensemble to get to know each other more quickly, which in turn could make rehearsing more effective. As the participants experienced a state of flow during the sessions, this can help them to function at full capacity (Csikszentmihalyi et al., 2005:230), which can also save time in rehearsals.

Lastly this article adds to the field of research on the experiences of performers who use Dalcroze Eurhythmics to prepare for performance and thus addresses the last aspect of the research problem. Connecting the pedagogy of Dalcroze Eurhythmics with the flow experience is a current and timely topic that was recently discussed at the 2016 national conference of the Dalcroze Society of America (American Orff-Schulwerk Association, 2016), where the theme of the conference was “Flow in performance: theories/practices.” In a recent publication of Le Rythme, Großberger (2019, p. 177–178) explored teaching strategies to initiate flow during her Eurhythmics classes for adults. She compared the characteristics of flow with the action fields of Eurhythmics. However, as far as we are aware, this study might be one of the first to link the lived experiences of Dalcroze participants, through an IPA study, to the theory of flow. Custodero’s (2005) article examines the flow experiences of young children in music learning environments that include Dalcroze classes as part of the music learning environment, among others. Custodero (2005, p. 188), however, uses an observational method to look for flow indicators in young children and followed the method of grounded theory (Custodero, 2005, p. 189).

Recommendations for possible future research would be to look into the aspect of “shared flow” that ensembles may experience when performing and rehearsing together. Nakamura and Csikszentmihalyi (2002, p. 259) mentioned that in research on the flow experience the focus was mostly on the individual and consequently there is room for further investigation of the concept of “shared flow.” In this regard, Habe et al. (2019, p. 1) stated that “Elite musicians and top athletes experienced flow more often in group than in individual performance settings”. In an ensemble performers are working toward achieving a unified goal. In that sense, the concept of “shared flow” can be linked to successful ensemble playing and thus to our findings. We contend that the ensemble will have experienced a state of “shared flow” during the Dalcroze sessions and thus will have experienced all the benefits of this state of flow. The experience of flow can also be studied in other Dalcroze Eurhythmic settings and it would be interesting to do a similar quantitative intervention study, where measurements and performances before and after the Dalcroze sessions with the ensemble can be recorded and analyzed.

As the Dalcroze sessions helped the ensemble on different levels and led to overall heightened awareness, improvement of relationships and consequent improvement in musicianship, it could be a significant approach to add to ensemble preparation. The Dalcroze sessions were also enjoyable, which can contribute to the general wellbeing of an ensemble. The findings can inform the teachers’ own pedagogy in teaching ensemble playing or ensemble courses. This study could be used to advocate for the inclusion of the Dalcroze approach in ensemble courses at university level. Furthermore, the study links Dalcroze Eurhythmics with the “Flow experience” directly, which is a current topic. This theoretical link can contribute to the inclusion of Dalcroze Eurhythmics in different ensemble and general courses.

## Data Availability Statement

The raw data supporting the conclusions of this article will be made available by the authors, without undue reservation, to any qualified researcher.

## Ethics Statement

The studies involving human participants were reviewed and approved by the Ethics Committee of the Faculty of Arts (REC) of the North-West University. The patients/participants provided their written informed consent to participate in this study.

## Author Contributions

CW is the main author and corresponding author. She did the data collection and wrote the manuscript. She participated in the workshops as a performer. LV also participated in the Dalcroze Eurhythmics workshops as a performer, supervised the study, helped with the conceptualization of the manuscript, assisted with data analysis, reduced the manuscript from a longer text, and edited the manuscript.

## Conflict of Interest

The authors declare that the research was conducted in the absence of any commercial or financial relationships that could be construed as a potential conflict of interest.

## Footnotes

1 Dynamic Rehearsal is a method developed and named by Greenhead and therefore it is written with capital letters.

2 Pseudonyms were used for the participants.

3 The two Dalcroze teachers have a Dalcroze License qualification and a Diplôme Supérieur Jaques-Dalcroze, respectively. One teacher has 30 years’ and the other 20 years’ experience in teaching Dalcroze Eurhythmics at different levels and in different contexts.
