# Supplementary material for: Exploring the Lived Experiences of Instrumental Ensemble Performers With Dalcroze Eurhythmics: An Interpretative Phenomenological Analysis
Source: Front Psychol. 2020 Mar 3;11:336. doi: 10.3389/fpsyg.2020.00336 (PMC7063099; doi:10.3389/fpsyg.2020.00336)
Supplement: Supplementary file 1 [file Table_1.DOCX]

**Online supplementary material – Table A**

**Dalcroze activities during different sessions**

| **Session 1 (60 minutes) – Instructor 1** | |
| --- | --- |
| Activity 1a  Activity 1b | Walk in any direction (without any music). Imagine that there is an eye on your chest. Imagine that you are opening the eye and looking at the people around you. Close the eye again and go back into your own bubble.  While still walking in the room, try to establish the same rhythm between everybody (without music). If everyone is walking to the same rhythm, anybody can stop at any given moment and then everybody has to stop. Then anybody can start again at a new speed and everybody has to start again and follow this new speed. |
| Activity 2 | Walk to the pulse of the music being played. The instructor stops the walking and instructs everyone to show the pulse of the music in their shoulders. Afterwards everybody walks to the pulse again; after a while the instructor stops the walking again and then everyone puts the pulse in their heads. This pattern continues with putting the pulse in the hips, knees and feet every time that the instructor stops the walking. The last time the instructor stops the walking everyone can decide for themselves in which body part they want to put the beat. |
| Activity 3 | Everybody forms a circle and claps on the pulse of the music being played. Then everybody just claps on one beat as the pulse moves from person to person in the circle. Anybody can change the direction of the pulse at any time, but you have to make sure that you indicate the change of direction very clearly through eye contact and your body language. Then you can hand over the pulse to anyone in the group, but again you have to make sure that you indicate clearly, through eye contact and body language to whom you are passing the pulse. |
| Activity 4 | The instructor plays the complete “Charleston” and everyone can perform any free movements to the “Charleston” |
| Activity 5 | Sit on the ground and close your eyes. Listen to the opening melody and try to portray the musical line with your hands. Now just listen to the cello part and portray the melodic line of the cello with your hands. |
| Activity 6a  Activity 6b | Portray the opening of the “Charleston” through improvisation and free movement. When the opening is repeated portray the cello part with free movement and when it is repeated again portray the bassoon part with improvisation and free movement.  Stand in pairs. One person portrays the cello part and the other person portrays the bassoon part through improvisation. Change partners and do the same exercise again with the new partner. |
| Activity 7 | Portray the emotion of this introduction with the cello and bassoon through free movement and improvisation. |
| **Session 2 (90 minutes) – Instructor 2** | |
| Activity 1 | The instructor plays one voice, two voices or full chords on the piano. When you hear one voice, you walk alone, when you hear two voices you find a partner and walk in pairs. When you hear full chords on the piano everybody forms a train and walks together in a line following the person in front. |
| Activity 2a  Activity 2b  Activity 2c  Activity 2d  Activity 2e | Everybody invents their own interesting and characteristic walk, with your own rhythm in mind (without music).  Continue with your characteristic walk and vocalise your specific walk with any sounds that will suit your specific walk.  At a specific signal from the instructor you start walking next to somebody and maintain your own characteristic walk and sound.  At another signal from the instructor, you and your partner will start morphing your walk and sound into a single uniform sound and walk. At another signal from the instructor the pairs will merge and form a quartet that will morph into a single uniform sound and walk.  The instructor will give another signal and then the quartets will stop the sound but continue with the walk in silence, maintaining the rhythm. At another signal you will stand still and internalise the walk and the sound. When the instructor gives another signal you will start walking again. At another signal you will start adding the sound again. |
| Activity 3a  Activity 3b  Activity 3c | Everybody gets a scarf. The instructor played fast recorded music, not the Charleston or Tango. You can experiment with different methods of travelling and moving with the scarf. The scarf should stay on your body, arm, back etc. The speed that you are travelling and moving with the scarf, will help the scarf to stay on your body. Keep close contact between your body and the scarf. Think about flying with the scarf. You can travel in any direction and through the whole room with the scarf, but be aware of everybody spatially.  The instructor plays the same recorded music as with the scarf. There are one ball within the six participants. Try to travel and move in the same way as you did with the scarf and pass the ball seamlessly from one person to another person while traveling in the room. Think of the ball as the melodic line.  The instructor plays another piece of recorded music. You travel through the room again, passing the ball around from person to person, but this time you can decide where would be a suitable place in the music to pass the ball to a new person. |
| Activity 4a  Activity 4b  Activity 4c | The instructor hands out a piece of paper and a marker for every member of the ensemble. See the piece of paper as the floor of the room or a stage. The instructor has divided Martinů’s “Tango” movement into 5 smaller sub-sections. Listen to the sub-sections of the “Tango” and draw the way in which you see the floor plan/stage plan for that section of the music. Who are the personalities or characters (instruments) on stage? Where are they on stage in relation to the audience and to each other? What is foreground and what is background? Who is interacting with whom? Instructor plays the first sub-section twice and you draw the stage plan as you see it.  Everybody discusses how they see the floor plan and then we implement this as a “stage production” of the opening sub-section of the “Tango”. Everybody portrays their own instrument.  Activities 4a and 4b are repeated with the second sub-section of the “Tango” afterwards.  Continue with the choreography of the Tango section by section and perform the complete choreography in the end. Every person is portraying his/her own instrument in the choreography. |
|  | **First round of interviews are conducted** |
| **Session 3 (120 minutes) – Instructor 2** | |
| Introduction | Discuss the background of the “Tango” by Martinů  Also do some general warming-up exercises to recorded music |
| Activity 1 | Everybody gets a white mask to put on. The instructor plays a medley of different styles of recorded music. Everyone portrays the character of the music through improvisation and free movement while wearing the mask. The mask provides a form of anonymity. |
| Activity 2 | Instructor plays the complete “Tango” and everyone does improvisation and free movement to the “Tango”. Try to think about the story of “Tango” that was discussed in the Introduction to the session. |
| Activity 3 | The instructor starts a story and stops at a point and the next person goes on with the story. Everyone gets a chance to add a part to the story from the point where the previous person stops. |
| Activity 4 | Move to the instruments and play through the “Tango” once. |
| Activity 5 | Follow the instructions through movement. The first instruction is just to walk to the pulse of the music being played on the piano by the instructor. The next instruction is to walk twice as fast as the pulse and after that to revert to the original pulse. Now go twice as slowly as the pulse and then revert to the original pulse. The next instruction is to go three times as fast as the pulse and then back to the original pulse. The instructor then varies walking twice as fast as the pulse and walking three times as fast as the pulse. Shifting constantly between these two rhythms. |
| Activity 6a  Activity 6b  Activity 6c  Activity 6d  Activity 6e | Stand in the circle. Walk three pulses with your feet in the following way: Right foot steps to the left crossing the left foot, then left foot, which hasn’t moved, makes a step in the middle and then the right foot steps back to where it began next to the left foot. This is three pulses. Repeat this pattern to the other side for the next three pulses. While doing this clap hands and snap two even pulses with the fingers. Clap on the first beat and snap between the second and third beat in the feet.  Continue with this pattern in the feet and hands of three against two. Now stop the clap on the first beat and only do the snap between beats two and three. Then start vocalising the two beats instead of clapping hands and snapping fingers on them.  Switch this pattern around and now walk the two beats and clap the three beats with the hands. Practise this.  Switch between the two patterns of activities 6a and 6c. Do four repetitions of the three with the feet and two with the hands and then change to four repetitions of two with the feet and three with the hands. To break this activity down further, just do the four repetitions of the three pulses with the feet and then four repetitions of the three pulses with the hands. Repeat this. Now do the same with the two pulses and after that try to do everything together again in repetitions of four. Look at each other while doing this and make sure that you are executing the moves together as a group. Make sure to make eye contact with everyone in the ensemble.  The instructor plays either the three-pulse pattern or the two-pulse pattern on the piano and everybody walks to the pattern that is being played on the piano. After this you have to walk the opposite pattern than the one being played on the piano. If the piano plays the two-pulse pattern, you have to walk the three-pulse pattern and if the piano plays the three-pulse pattern you walk the two-pulse pattern. |
| Activity 7a  Activity 7b  Activity 7c  Activity 7d | Everyone picks up their instruments again. The instructor performs the three-against-two pattern on the piano and everybody decide themselves whether they want to perform the three-pulse pattern or the two-pulse pattern on their instruments. Just use the notes DEGA from the pentatonic scale.  Listen to each other. Try to hear which instruments are playing which patterns. Follow the tempo indicated by the movements of the instructor (it can change). Close your eyes and listen to each other and make sure that you are together.  Take notes from the “Tango” that are either triplets or in the two-pulse pattern. Everyone decides themselves whether they want to perform the two-note pattern or the three-note pattern. When the instructor gives a signal you have to change to the other pattern.  The three-note and two-note patterns are now played a bit faster. If the instructor points at an instrument, only that instrument should play the pattern that he/she was playing and everybody else is silent. After this everybody falls in again with the pattern they were playing. The instructor can sometimes point to two people and then those two should continue playing their pattern and everybody else should be silent. |
| **Session 4 (90 minutes) – Instructor 2** | |
| Activity 1a  Activity 1b | As an ensemble we have to decide how we want to interpret the two opening chords of the “Tango”. Everybody is given a ball and you can decide yourself how you feel the two opening chords. Each person will portray their own version of these opening chords through bouncing and catching the ball, or through any other movement with the ball. Each person shows their interpretation to the rest of the ensemble and then everybody else copies that person’s interpretation. Also add vocalisation to your own version.  Everyone picks up their instruments. The instructor performs the different versions with the ball and the ensemble plays these different versions. We have to be together and follow the actions that the instructor performs with the ball. In the end the ensemble decides whose version they liked the most and practises that version. |
| Activity 2 | Play through “Tango” again and work in detail on the playing of the “Tango” and the different parts. This is done while playing the piece on the instruments. The instructor focuses especially on the people who do not know the work very well, helping them get their rhythms and notes correct. The cello has a big solo in the beginning of the “Tango”. The pianist goes and taps the piano rhythm on the cellist’s back. This helps the cellist to feel the pulse and rhythm of the piano better and to play this solo rhythmically more correctly. |
| Activity 3 | Talk about how the ensemble sees and feels the characteristics of the different instruments in this “Tango”. |
| **Session 5 (100 minutes) – Instructor 2** | |
| Activity 1 | Play through the “Charleston” |
| Activity 2a  Activity 2b  Activity 2c | Divide into two groups of three people. Stand opposite each other in the room in two rows of three people each. The instructor plays a recorded track of “Take Five”. We are going to walk towards each other, but you can only walk on the pulses that the instructor calls out. Make a definite and precise movement on the pulse that you should walk on and freeze on the pulses that you shouldn’t walk on. It should be a very crisp and definite movement that you make when walking on the pulse that is called out.  Stand in a circle facing each other. Swing one arm for the duration of five pulses. Pulse one is when the arm reaches the lowest point. Vocalise the pattern instead of counting. Put a sound on the upbeat and keep the downbeat silent, but still continue swinging the arms on the five pulses.  Start by repeating activity 2a, but now the instructor calls out more than one pulse that you should walk on. |
| Activity 3a  Activity 3b  Activity 3c  Activity 3d | Stand in pairs of two. You and your partner decide on eight quick movements. Put these movements into a specific order from one to eight and practise this sequence. The instructor plays 8 pulses on the piano and calls out one number from one to eight. You do your whole pattern of eight movements pianissimo and quietly, but the movement on the number that the instructor has called out is fortissimo and big. You can even add a vocalisation.  We just start by doing the whole sequence of eight movements. Then you put accents on movement 4 and 7. After that you put accents on movement 2, 4 and 7.  Do the following eight-bar pattern with these eight movements. 1 bar consists of one repetition of these eight movements. Put accents on the following pulses in each bar.  Bar 1 – Pulse 4 and 7  Bar 2 – No accents  Bar 3 – 2, 4 and 7  Bar 4 – No accents  Bar 5 – 7  Bar 6 – 5  Bar 7 – 1, 4 and 7  Bar 8 – 2 and 5  Practise this whole pattern with the movements.  Stand opposite your partner in the room. You are going to walk towards each other using the eight-bar pattern described in activity 3c. You can only step on the given pulses in each bar. Everybody calls out the numbers on which they are allowed to step in this eight-bar pattern. |
| Activity 4a  Activity 4b | Pick up the instruments again. Every instrument chooses one note or a chord to play the accented notes in the eight-bar pattern described in activity 3c. You only play on the pulses given.  Play the section in the “Charleston” from where this particular accent pattern originates at half the tempo. Really focus on the places in which you should put the accents. Repeat this section a bit faster and lighter. |
|  | **Follow-up interviews are conducted** |

**Online supplementary material – Table B (Cross-case Analysis)**

| **Themes** | **Emma’s categories** | **Cathy’s categories** | **Anne’s categories** | **Peter’s categories** | **Judy’s categories** | **Mary’s categories** | **Benjamin’s categories** |
| --- | --- | --- | --- | --- | --- | --- | --- |
| **1. Heightened awareness of music time and space** | **Awareness**  1.Time  2.Space  3.Heard other parts differently  4.Own role in ensemble | **Awareness**  1.Time  2.Space  3.Interaction between parts  4.Get to know and understand the role of different instruments  5.Visual stimuli distracts from listening  6.Accents  7.Moods of other ensemble members | **Awareness**  1.Time flies when actively involved  2.Usage of space  3. Became aware of interaction between instruments.  4.Give fresh perspective 5.Enhance focus  6.Holistic awareness | **Awareness**  1.Time: Felt long  2.Space: Used whole room  3.Role of different instruments  4.Fresh perspective on music  5.Body when playing cello  6.Dynamics  7.Rhythm and tempo | **Awareness**  1.Time flies  2.Interesting usage of space  3.Interaction between instruments  4.Own role in ensemble  5.Intense focus  6.Holistic awareness  7.Many different ways to practise | **Awareness**  1.Time  2.Space  3.Other instruments  4.Own part in relation to others  5.Movement in music  6.General phrasing and characters of instruments | **Awareness**  1.Became aware of each other |
|  | *“Let me explain it like this: if the challenge meets the ability, then time flies; if the challenge exceeds the ability, then time slow down. So where I struggled, it felt like everything was going in slow motion and then it wasn’t a nice place for me to be in. So where it was easy the time flies and where it was difficult it felt like forever.”* | *“This approach can really help the ensemble to become more aware of each other’s parts and the relation between the different instruments from the beginning of preparation. It helps if you do not only learn your part in the beginning, but you immediately learn your own part in relation to the other parts.* | *“...for that hour and a half that we worked together...you were so focused on what was going on. There were no time or energy or whatever to think about what was going on outside the room beforehand.”* | *“I think one thing that it made me aware of again is my whole body and if you sit behind your cello and play, you should still play with your whole body and you can forget this easily. [...] When I sat behind my cello again (after moving), it was a different feeling than when I usually sit behind my cello and play.”* | *“In the first session you were really… it depends on the activity. You were kind of like moving in your own space and sometimes with a friend so your space was limited because you were sharing with so many people.[…] but in the second session, because we were moving together as a whole group, we had so much space to work with.”* | *“You know you listen to a piece in one way, you know only in relation to your own instrument and with both these sessions, you were made aware of the other instruments and understanding their melodies better.”* | *“Again I come back to the ball activity…at the ball activity I would say nearly a 100% (aware of each other) because we all saw that ball, we were all interacting and there I felt we did work together and we were aware of each other much more so in that.”* |
| **2. Beneficial for relationships in ensemble** | **Interpersonal relationships**  1.Can trust each other  2.Get to know each other quickly  3.Figure out interaction between ensemble members  4.Conflict resolution | **Connection**  1.Build relationships and trust  2.Broke tension | **Bonding**  1.Bonding quickly  2.Good icebreaker  3.Get to know each other better  4.Promote group dynamics | **Group dynamics**  1.Felt comfortable faster  2.Get to know each other quicker  3.Enjoy different interactions | **Interaction**  1.Intuitively connect and bring people together  2.Choreography as a group  3.Positive to move in different combinations  4.Different views on interpretation  5.Help each other  6.Respects partner in movements | **Communication**  1.Bonding quickly in ensemble  2.Good icebreaker in ensemble  3.Different reactions from different people  4.Conflict resolution and commitment  5. Communication is a challenge in ensemble playing, but Dalcroze supports it  6.On a musical level  7.Less uncomfortable in group work | **Benefit**  1.Teamwork |
|  | *“The interaction between the players changes your perspective and I saw the people in a different light and also I got to know them and I think as a group we also got to know each other and get a feeling for each other. This might make the first uncomfortable rehearsal a bit easier.”* | *“In ensembles I also think that this approach could help to build relationships and trust and this is something that I think could really help an ensemble to click better and really make beautiful music when playing together.”* | *“I was glad to see everybody again. So in the second session I realised that there was already a bond between us and I think that is extremely important in chamber music or any type of performance where you are more than one.”* | *“This type of work makes me feel comfortable faster, because you have to expose yourself quicker.”* | *“I think firstly as I said before, it’s the fastest way to get to know the people that you’re playing with in a really deep deep sense. As I said it’s very kind of like an intuitive [feeling]. I really feel that you always have that deep connectedness among people that you shared Eurhythmics class, but I think that aspect could really help ensemble players to quickly bond and quickly come together to create something.”* | *“There’s communication as a group, there’s communication between the different individuals, there’s communication from small group towards the bigger group and I think at this stage, it felt like that form of musical communication was supported by the whole Dalcroze approach. So by the time we will be playing this, I know we have to come in at a certain entry and that communication was supported by Dalcroze.”* | *“In the playing together you’re trying to be correct and until you’ve developed that team work you’re probably not paying a hell of a lot of attention to the other people, but this kind of activity I think could build the team work that would make the musical attention to each other happen more quickly.”* |
| **3. Improved musicianship** | **Improvement**  1.Ensemble interpretation and playing  2.Clarity in ensemble playing  3.Technique and musicianship  4.Playing less inhibited  5.Visual movement stimuli improved sound and playing | **Benefit**  1.Musical understanding of piece  2.Cross-rhythms and togetherness in ensemble playing  3.Self-confidence as a musician  4.Piano touch | **Influence on preparation**  1.Got holistic picture of ‘Tango’  2.Could hear difference after movement activity  3.Later less self-conscious  4.Improved listening | **Benefit**  1.Musical understanding  2.Knowing how to practise  3.Help overcome shyness  4.Playing more relaxed and better  5.Europe more than South Africa | **Improvement**  1.Understand music better  2.Internalising music  3.Improvement of musicianship  4.More focused analytical listening  5.Playing improved through movement  6.Sound production improved through movement  7.Movement vocabulary | **Awareness**  1.Sharpens listening | **Benefit**  1.Togetherness in ensemble |
|  | *“I think Dalcroze helped to develop me as a musician. […] So it deepened my experience of the music, it expanded my expressive possibilities and it reduced my inhibitions and I also think it improved things like sound…”* | *“As a musician, Dalcroze had a big influence on my musicianship. I am reserved, as I already mentioned, and Dalcroze helped me to open up as a musician and free my body more when performing.”* | *“...and you could definitely see, if we went back to the instruments that there was a big difference. So that was very positive for me....it was almost like a before and after.”* | *“Everything I did with Dalcroze so far and all its derivatives with subjects like aural training and so, just to move to the music helps immensely in the way you understand the music.”* | *“The most important thing for me, is that it teaches you what to listen for, it teaches you who to listen for, because the general thing is that you should listen, you should play together with other people and it makes you aware of how you should do something and how you as a group should do it...”* | *“I’m definitely more sensitive to what she does or he does, my pianist, and that’s because of Dalcroze. […] It definitely sharpens your listening and your awareness of other musicians.”* | *“I come back again to just the togetherness on the start and finish that we did with the ball activity. Just to make sure we start together, that’s the only thing I would focus on (as being beneficial).”* |
| **4. Enjoyment and wellbeing** | **Wellbeing**  1.Gives energy and inspires me  2.Freedom  3.Enjoy interaction and group work the most | **Enjoyment**  1.Gives energy  2.Increase with familiar activities  3.More inspired when moving with others  4.More comfortable in group  5.Rhythm activities | **Enjoyment**  1.Moving to the music  2.Increased with sessions  3.Interaction between ensemble members  4.Group work more  5.Exercises with equipment | **Fun**  1.Lifts your mood  2.When it felt like a game  3.Enjoyment increases when there is a goal  4.Moving together  5.Combining movement and playing instruments  6.Get rhythm into body | **Positive experience**  1.Improved mood  2.Moving freely  3.Enjoyed group work more  4.Activities with different materials  5.Moving and then playing | **Wellbeing**  1.Focus on music is therapeutic | **Positive experience**  1.Enjoyed interacting with people in ensemble |
|  | *“The thing that I enjoy the most about Dalcroze is the social interaction, so I enjoy things like mirror activities and, like playing in an ensemble, to feed off each other…So I look for that ensemble playing in movement and to improvise together. That is when I have my peak experiences with Dalcroze.”* | *“Sometimes I would feel like I am too tired to move and that I would rather just sit for a moment, but when I start moving, I start enjoying it and it is like I get new energy.”* | *“When I compared the two sessions after I enjoyed the second session so much, I realised that we worked more in the group in the second session.”* | *“I liked the structured things that we did, like when we had to use the ball to find the first pulse in the ‘Tango’ or where we had to improvise movements on that series of numbers that we walked on. The things where we have a goal, I enjoyed the most.”* | *“It’s a fun way of learning you’re not just like sitting and oh, so the cello comes in at bar five, so I have to make sure that I’m quiet so that they can hear the cello blah blah blah. No, you kind of like … instead of just seeing that on paper, you see that visually in a really cool manner if you know like we did with the Dalcroze teacher. We acted out, you know, we moved it out.”* | *“If I was having a bad day...it takes you a little bit out of your situation and it removes you from your own reality, […] You are forced to leave whatever negative or positive or what issues you have concerning your good or bad day and you are focused on listening to the music and to react to that and that is I think very good therapy for getting your mind clean.”* | *“It impacted positively because I loved just to be relaxed and to communicate very easily. […] I felt it was a relaxed group because we all knew each other.”* |
| **5. Informing pedagogy of Music educators and Dalcroze practitioners** | **Pedagogy**  1.Best way to learn through movement  2.Self-conscious when focus on individual and correctness  3.You must experience Dalcroze to understand it  4.Will not work for everybody  5.Power role of educator affects own experience (teacher-centred vs. learner-centred)  6.Interaction changed  7.Prefer visual stimuli | **Pedagogy**  1.Kinaesthetic learning important  2.People watching causes insecurity  3.Personal preference important  4.Fun way to learn  5.Saves time in rehearsals  6. Valuable approach | **Influence on preparation**  1.More for first time players  2.Feasible method | **Pedagogy**  1.More beneficial for amateur ensemble  2.Better progression for music beginners | **Pedagogy**  1.Improved as music educator  2.Challenging but not discouraging | **Pedagogy**  1.Feasible approach  2.Must experience Dalcroze before judging it  3.Instructors made us comfortable | **Struggle**  1.To pay attention to rest of the ensemble  2.Reacting correctly  3.Didn’t learn anything and doubts value of approach |
|  | *“If the facilitator handles the sessions in a learner-centred way and the emphasis isn’t on the performance or correctness or something like that, that doesn’t put the spotlight on the individual, then I feel uninhibited and free and I can enjoy it and I feel better afterwards.”* | *“I think the music world would be empty without Dalcroze. It really puts a new dimension of enjoyment in music education. It is such an inspiring way to learn music and it is also such a logical way in which to learn music.”* | *“I think the big difference was…look she worked a lot with Peter and Judy, they didn’t know the parts as well as we did. So just to watch that, I could really see the value of learning a piece of music through Dalcroze.”* | *“This is my view, but I think the more amateur the people in the ensemble are, the more definite the difference will be (after a Dalcroze session). I think a professional trio might also benefit from something like this, but it wouldn’t be so obvious. […] So I think it is a very feasible approach, especially in an educational setting.”* | *“…in general I think that was great for me not only as a musician but also kind of like a music educator and to see how they approach the same method but in a different ways.”* | *“I thought she was very good at making us feel comfortable and doing little exercises bringing you eventually to a point […]. By that time you were actually quite comfortable and open to the new ensemble and new little things being done.”* | *“When we had to move our feet the lady asked us every time to move with the right foot first and in dancing for a man that’s the opposite way round. If you’re in the army and you’re marching it is left foot first. So if I had to choose one that I remember very clearly I was always being caught wrong-footed.”* |
| **6. Social and cognitive challenges** | **Challenge**  1.Struggle to integrate playing and moving  2.To feel music instinctively after analysing  3.Felt confused when we had to draw | **Frustration**  1.Couldn’t apply all the activities in playing  2.Self-conscious with free movement  3.Not everybody knew their notes  4.Distractions | **Self-consciousness**  1.New to Dalcroze  2.Felt exposed when moving alone  3.Didn’t enjoy initially | **Frustration**  1.Confusing if too abstract  2.Dancing freely  3.Not knowing the goal | **Intense**  1.Workshop and instructor was really intense | **Uncomfortable**  1.Body issues | **Separates music and movement**  1.Separates dancing and music  2.Separates playing instruments and movement  **Struggle**  3.Proper breathing after movement  4.Not to bump into people  5.Physically sore |
|  | *“I struggled to merge the playing and the movement, because I know what to do with my instrument and have certain strategies I follow to do things. The movement was almost like a new strategy and I couldn’t merge the movement strategy with the playing fast enough, because I did it differently my whole life.”* | *“It sometimes frustrated me that everybody wasn’t on the same level regarding knowing the notes of the piece. The result of this was that we had to pause very long at certain sections, so that some people could get the notes and rhythms correct. I think it felt like wasting time for some people.”* | *“…I felt so exposed, even though we moved in the group, the focus was for me very much on the individual and I was very unsure of myself. So that was a difficult session for me.”* | *“I do not like the part of Dalcroze where you have to move around randomly and freely and try to portray what you hear. I feel it is nice and everything, but I am not a contemporary dancer and it doesn’t really help me to understand the music.”* | *“It was an intense day and especially because it was after the rest of the weekend workshop. The instructor was also very intense person. She expects a lot from you, she really pushes you, which I think is good, but sometimes it was just too intense for me.”* | *“…so initially for me it was actually a bit uncomfortable, but that’s not criticism to anybody; its criticism to myself and my own awkward issues with my body. It’s nothing specifically negative towards Dalcroze.”* | *“And I found the movements themselves became something to have to learn; in other words I didn’t fall naturally into the movements that we we’re asked to do. So in that respect it was negative experience because in my mind it took my mind away from the music. […] So it took it away completely from, to my mind, the objective of the thing.”* |
